# Taxonomic revision of the Long-tailed Mole (Talpidae: *Scaptonyx*) with description of a new species from the Gaoligong Mountains

**DOI:** 10.1093/jmammal/gyae142

**Published:** 2025-03-15

**Authors:** Wen-Yu Song, Zhong-Zheng Chen, Quan Li, Wen-Hao Hu, Hong-Wei Zhou, Meng-Ru Xie, Xue-You Li, Xue-Long Jiang

**Affiliations:** Key Laboratory of Genetic Evolution and Animal Models & Yunnan Key Laboratory of Biodiversity and Ecological Conservation of Gaoligong Mountain, Kunming Institute of Zoology, Chinese Academy of Sciences, Kunming, Yunnan 650204, China; Vector Laboratory, Institute of Pathogens and Vectors, Yunnan Provincial Key Laboratory for Zoonosis Control and Prevention, Dali University, Dali, Yunnan 671000, China; Key Laboratory of Genetic Evolution and Animal Models & Yunnan Key Laboratory of Biodiversity and Ecological Conservation of Gaoligong Mountain, Kunming Institute of Zoology, Chinese Academy of Sciences, Kunming, Yunnan 650204, China; Collaborative Innovation Center of Recovery and Reconstruction of Degraded Ecosystem in Wanjiang Basin Co-Founded by Anhui Province and Ministry of Education, School of Ecology and Environment, Anhui Normal University, Wuhu, Anhui 241002, China; Key Laboratory of Genetic Evolution and Animal Models & Yunnan Key Laboratory of Biodiversity and Ecological Conservation of Gaoligong Mountain, Kunming Institute of Zoology, Chinese Academy of Sciences, Kunming, Yunnan 650204, China; Gaoligong Mountain Forest Ecosystem Observation and Research Station of Yunnan Province & Dulongjiang Forest Ecosystem Research Station, Kunming, Yunnan 650223, China; Key Laboratory of Genetic Evolution and Animal Models & Yunnan Key Laboratory of Biodiversity and Ecological Conservation of Gaoligong Mountain, Kunming Institute of Zoology, Chinese Academy of Sciences, Kunming, Yunnan 650204, China; Collaborative Innovation Center of Recovery and Reconstruction of Degraded Ecosystem in Wanjiang Basin Co-Founded by Anhui Province and Ministry of Education, School of Ecology and Environment, Anhui Normal University, Wuhu, Anhui 241002, China; Vector Laboratory, Institute of Pathogens and Vectors, Yunnan Provincial Key Laboratory for Zoonosis Control and Prevention, Dali University, Dali, Yunnan 671000, China; Vector Laboratory, Institute of Pathogens and Vectors, Yunnan Provincial Key Laboratory for Zoonosis Control and Prevention, Dali University, Dali, Yunnan 671000, China; Key Laboratory of Genetic Evolution and Animal Models & Yunnan Key Laboratory of Biodiversity and Ecological Conservation of Gaoligong Mountain, Kunming Institute of Zoology, Chinese Academy of Sciences, Kunming, Yunnan 650204, China; Gaoligong Mountain Forest Ecosystem Observation and Research Station of Yunnan Province & Dulongjiang Forest Ecosystem Research Station, Kunming, Yunnan 650223, China; Key Laboratory of Genetic Evolution and Animal Models & Yunnan Key Laboratory of Biodiversity and Ecological Conservation of Gaoligong Mountain, Kunming Institute of Zoology, Chinese Academy of Sciences, Kunming, Yunnan 650204, China; Gaoligong Mountain Forest Ecosystem Observation and Research Station of Yunnan Province & Dulongjiang Forest Ecosystem Research Station, Kunming, Yunnan 650223, China

**Keywords:** endemism, Hengduan Mountains, insectivores, mountain biodiversity, mountains of southwest China, river barrier, 横断山, 江河阻隔, 山地生物多样性, 食虫类, 特有动物, 中国西南山地

## Abstract

*Scaptonyx fusicauda* Milne-Edwards, 1872, is a mole species and a burrowing animal occurring from central China to northeastern Myanmar and northern Vietnam. This is the only extant species currently known in the genus *Scaptonyx* (Talpidae), but recent studies have revealed highly diverse lineages within this taxon. However, the broken type specimen and unspecified type locality of this species have hindered comparison between specimens towards a taxonomic evaluation. We reviewed the literature documenting the expeditions of the collector Armand David and narrowed down the type locality of this species to Minshan Mountain, western Sichuan, China. We compared *S. fusicauda* topotypes with specimens from 2 separated mountain ranges in western Yunnan, China—*S. f. affinis* Thomas, 1912 from the Baima Mountain and an undescribed species (*S.* sp. 1) from the Gaoligong Mountains. Specimens from the 3 localities can be distinguished by multiple external, craniomandibular, and dental characteristics. Phylogenies based on mitochondrial and nuclear genes also provided consistent topologies supporting *S. fusicauda*, *S. f. affinis,* and *S.* sp. 1 as distinct monophyletic species. *Scaptonyx* sp. 1 split from the sister clade (*S. fusicauda* + *S. f. affinis*) ca. 19.79 Ma, while *S. f. affinis* split from *S. fusicauda* ca. 9.56 Ma. Following these findings, we recognize *S. f. affinis* as a distinct species, *S. affinis*, and describe *S.* sp. 1 as a new species*. Scaptonyx fusicauda* occurs in the Sichuan Basin to the west, *S. affinis* from the mountains in the east of the Salween River to central China and northern Vietnam, and *S.* sp. 1 in the Gaoligong Mountains on the watersheds of the Irrawaddy and Salween rivers—encompassing western Yunnan, China, and northeastern Myanmar.

## Teaser

We reviewed the journey of Armand David from 1868 to 1869. On these grounds, we clarify the type locality of *Scaptonyx fusicauda* Milne-Edwards, 1872 within the vicinity of the Minjiang River source, Minshan Mountain, Sichuan, China. Based on our study of newly collected topotype specimens of *S. fusicauda*, we recognize *S. affinis* Thomas, 1912, previously treated as a subspecies of *S. fusicauda*, as a distinct species. We also describe a new species from the Gaoligong Mountains, *S. wangi*, based on morphological examinations and phylogenetic analyses. We provide an updated taxonomy of the genus *Scaptonyx*, along with ecological and distribution information for the 3 extant species.

## 摘要

长尾鼹*Scaptonyx fusicauda* Milne-Edwards, 1872是分布于中国中部至西南部、缅甸东北部和越南北部的一种小型鼹科Talpidae动物。该物种是长尾鼹属*Scaptonyx*的唯一现存物种。近年来的研究提示该种存在高度分化的支系。然而由于模式标本破损且模式产地不清限制了该类群的分类研究进一步开展。本研究回顾了模式标本采集者谭卫道 (Armand David) 的旅程, 最终确认该物种的模式产地为中国四川岷山地区的岷江源附近。我们比较了岷江源与中国云南西部白马雪山的*S. f. affinis* Thomas, 1912和采自高黎贡山的*Scaptonyx* sp. 1的标本, 发现这三个地点的标本可以通过多个外形、颅骨和牙齿特征区分。基于线粒体和核基因构建的系统发育树也显示它们之间出现了高度的分化。分歧时间分析显示*S.* sp. 1与姐妹类群 (*S. fusicauda* + *S. f.affinis*) 的分化时间约为19.79 Ma, 而*S. f. affinis*与*S.fusicauda*的分化时间约为9.56 Ma。根据这些发现, 我们将*S. f.affinis*提升为独立物种: *S. affinis*, 并将*S.* sp. 1描述为一新物种: *S. wangi*。*S. fusicauda*分布在四川西部的山地和高原地区; *S. affinis*分布在怒江以东至中国中部的山地和越南北部: *S.wangi*分布在高黎贡地区 (伊洛瓦底江和怒江分水岭), 包括中国云南西部和缅甸东北部。

The genus *Scaptonyx*Milne-Edwards, 1872 are small-sized moles of the family Talpidae, distributed mainly in southwestern China. Milne-Edwards (1872) established this genus based on a broken specimen of *S. fusicauda*Milne-Edwards, 1872 from the “confins du Kokonoor et du Sé-tschouan (= borders of Qinghai and Sichuan).” The holotype of this species arrived in Paris in a very poor condition, and the skull was so injured that Milne-Edwards (1872) could only illustrate the teeth (Allen 1938). The collector of this specimen was Fr. Armand David, a Lazarist missionary, natural history collector, naturalist, and correspondent of the Muséum d’Histoire Naturelle, Paris, who made 3 major zoological expeditions into the interior of China in the late 19th century and contributed considerably to the collection. A subspecies, *S. f. affinis*Thomas, 1912, was described based on a specimen collected 12 miles southeast of A-tun-tsi (presently Deqin), located in the mountain pass of the Baima Mountain, northwest Yunnan, China. So far, *S. f. fusicauda* and *S. f. affinis* are the only extant members currently recognized under the genus (Ellerman and Morrison-Scott 1951; Wilson and Reeder 2005; Kryštufek and Motokawa 2018).

According to Kryštufek and Motokawa (2018), *S. fusicauda* occurs from central China to northern Myanmar and northern Vietnam. The nominate subspecies *S. f. fusicauda* is distributed from west to central China, including northwest Sichuan highlands and the Qinling mountains. *Scaptonyx fusicauda affinis* occupies mainly the mountainous areas of southwest to central China, from northern Vietnam and Myanmar to Yunnan, Qinghai, and Guizhou Provinces, China (Kryštufek and Motokawa 2018). Anthony (1941) found an individual from northern Myanmar on the west slope of the Gaoligong Mountains defined by the watersheds of the Irrawaddy River and the Salween River and considered it to be different from *S. f. affinis* based on its longer tail and larger upper premolar. However, the observed differences could not be confirmed as a basis for separation into different races and ultimately assigned the individual under *S. f. affinis* due to geographical proximity (Anthony 1941). Hill (1962) confirmed that these variations were not unique after examining more specimens and placed the northern Myanmar specimens under *S. f. fusicauda*. Thus, the Gaoligong population, bordering China and northern Myanmar, has been identified as both *S. f. fusicauda* (Hill 1962) and *S. f. affinis* (Kryštufek and Motokawa 2018). Notably, Wang (2003) considered the Gaoligong population different from any known taxa and assigned them under “*S. f. gaoligongensis*”; however, this was a *nomen nudum* for lacking a formal taxonomic description (ICZN 1999). The findings, nonetheless, indicated the need for taxonomic reevaluation of the *Scaptonyx* population in the Gaoligong Mountains.

In the new millennium, the employment of molecular techniques has led to significant progress in understanding the evolutionary history of *Scaptonyx* spp. Despite that early study placed *Scaptonyx* under tribe Scaptonychini based on morphological comparison (Van Valen 1967), He et al. (2017) reunited this genus with *Dymecodon*, *Neurotrichus*, and *Urotrichus* after studying the phylogeny of the Talpidae family. This study also identified an ancient (mid-Miocene) split lineage within the genus *Scaptonyx*. Based on these findings, Kryštufek and Motokawa (2018) included genera *Scaptonyx, Dymecodon*, *Neurotrichus*, and *Urotrichus* in the tribe Urotrichini. Later, comprehensive phylogeographic and demographic analyses revealed deeply diverged lineages in the southwest mountains of China, further prompting a taxonomic revision of this monotypic genus (He et al. 2019). However, the broken type specimen and an ambiguous type locality made morphological and range comparison impossible. The exact *S. fusicauda* type locality falls anywhere along the thousands of kilometers in the bordering zone of Qinghai and Sichuan Provinces, whose administrative borders have also undergone substantial changes over the last one and a half centuries, especially in its eastern section (Fu et al. 2017).

Here, following a substantial review of the literature documenting the journeys of Armand David from 1868 to 1869, we confirmed that the holotype of *S. fusicauda* was obtained from north of the Minjiang River source on the western slope of Minshan Mountain (see Discussion section for a detailed information). This area is currently known as Songpan and Jiuzhaigou Counties in Sichuan Province. Specimens were obtained from within the vicinity during a recent field investigation. With the new topotype samples of *S. fusicauda*, we recognized the subspecies, *S. f. affinis* as a distinct species and described a new species of *Scaptonyx* from the Gaoligong Mountains based on morphological comparison and phylogenetic reconstruction using 1 mitochondrial gene (*CytB*) and 6 nuclear gene segments. We also performed divergence dating analysis based on concatenated mitochondrial-nuclear genes and determined the distributions of the recognized species based on a broadly sampled *CytB* phylogeny.

## Materials and methods.

### Specimen and sample acquisition.

**Fig. 1.**
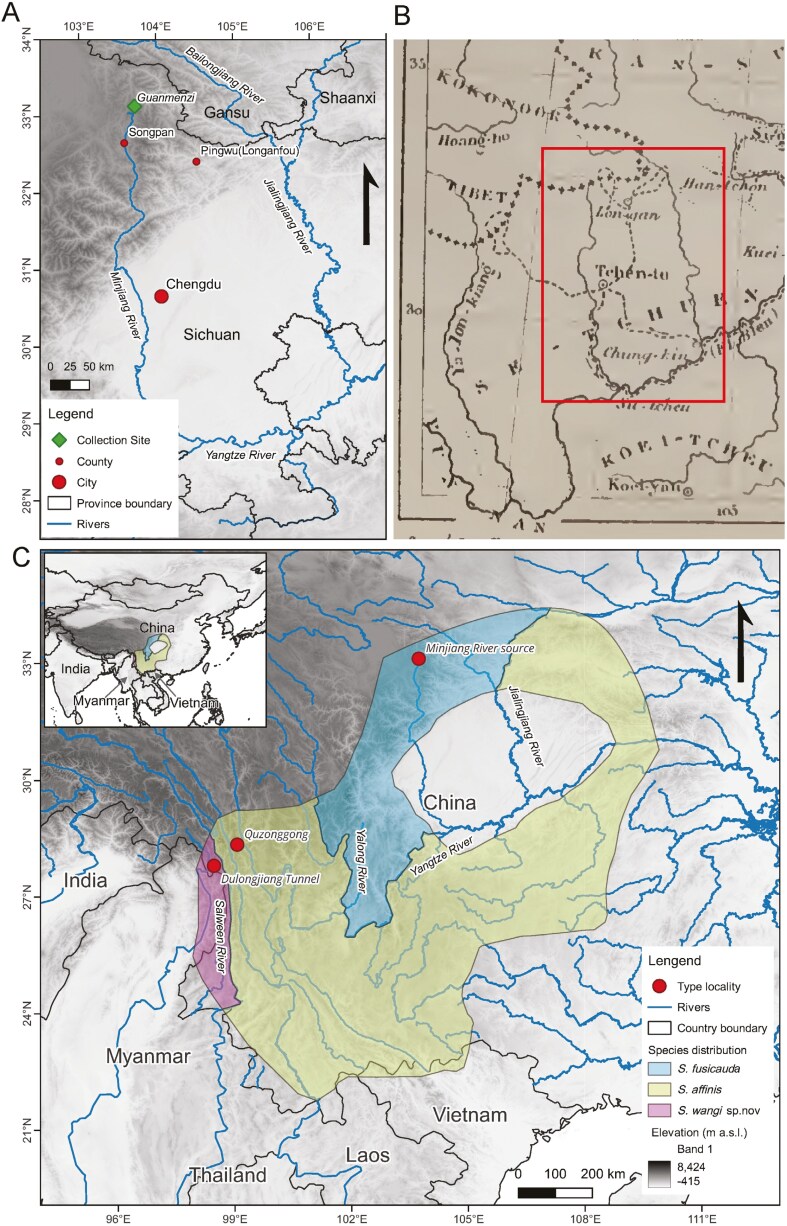
Collection sites and distribution ranges of the extant *Scaptonyx* species. A) Collection site of *S. fusicauda* topotypes; B) Route of Armand David visiting the “confins du Kokonoor et du Sé-tschouan” clipped from David (1875) with a red box framing the extent of A); Sichuan = Se-Tchuen, Gansu = Kan-Su, Chengdu = Tchen-tu, Pingwu = Longan; C) Distributions of the three recognized *Scaptonyx* species derived from the IUCN species distribution range of “*Scaptonyx fusicaudus*”.

Specimens and samples were acquired under the guidelines of the American Society of Mammologists on the use of wild animals (Sikes et al. 2016) and adhered to guidelines and regulations approved by the internal review board of the Kunming Institute of Zoology, Chinese Academy of Sciences. During a 14 day field investigation from June to July 2023 in the Minshan area, northwest Sichuan, we collected 2 *S. fusicauda* from Guanmenzi, 12 km north to the Minjiang River source on the border of Songpan and Jiuzhaigou with bucket pitfalls (Fig. 1). Nine *S. f. affinis* specimens obtained from Quzonggong, Baima Mountain, located approximately 20 km southeast of Deqin County between 2017 and 2023 were also examined. Seven specimens from Gaoligong Mountains were collected between 2018 and 2022. We assigned the Gaoligong specimens to *Scaptonyx* sp. 1 to distinguish them from those of other localities.

All specimens examined in the present study were prepared as traditional dried skins and cleaned skulls (Appendix I). The muscle and liver tissues were extracted and preserved in 99.7% ethanol in the field and stored in −80 °C freezers in the laboratory. The skins, skulls, tissues, and 95% ethanol-soaked carcasses were either stored at Kunming Institute of Zoology, Kunming, Yunnan Province, China (KIZ) or at the Institute of Pathogens and Vectors, Dali University, Dali, Yunnan Province, China (DLU). We also obtained morphological measurements of 5 *S. fusicauda* specimens from Minshan Mountain (Wanglang Nature Reserve) in the collection of the Sichuan Academy of Forestry, Chengdu, Sichuan Province, China (SAF).

### Morphological characterization.

Four external measurements, including head-body length (HB), tail length (TL), hind foot length excluding claws (HF), and body weight (BW), were taken from specimen labels or field notes for external morphological characterization (accurate to 1 mm for length and 0.1 g for weight). For craniomandibular delineation, 15 variables were measured using a digital caliper calibrated to the nearest hundredth (0.01) millimeter (Supplementary Data SD1). These measurements included: (i) Greatest length of skull, GLS; (ii) Palatal length, PIL; (iii) Postpalatal length, PPL; (iv) Cranial breadth, CB; (v) Interorbital breadth, IOB; (vi) Zygomatic breadth, ZB; (vii) Cranial height, CH; (viii) Upper toothrow length, UTL; (ix) Distance from the upper fourth premolar to the upper third molar, P^4^M^3^; (x) Maximum width across the upper second molars, M^2^M^2^; (xi) Foramen magnum breadth, BMF; (xii) Lower toothrow length not including first incisor, LTR; (xiii) Lower molars length, LLM; (xiv) Mandible length, ML; and (xv) Height of coronoid process, HCP (Supplementary Data SD2). Because Talpidae experienced various modifications in humeral morphology (Sánchez-Villagra et al. 2004; Piras et al. 2012; Sansalone et al. 2018), we compared humerus structures between the putative *Scaptonyx* species. Terminology for humeral descriptions followed Sansalone et al. (2016) and Costes et al. (2023). The description of pelage color was according to Werner’s Nomenclature of Colours (Syme 2018).

We employed one-way ANOVA analyses using SPSS v.19.0 (SPSS Inc., Chicago, IL, USA) to examine the differences in the 4 external and 15 craniomandibular measurements among the 3 putative *Scaptonyx* species. The Bonferroni correction test was applied to determine evidence of pairwise differences (*P* < 0.05 indicates the evidence was robust) between *S. fusicauda*, *S. f. affinis*, and *S.* sp 1. We performed principal component analysis (PCA) using PAST 4.13 (Hammer and Harper 2001) based on the correlation matrix to identify morphometric variation between *S. fusicauda* (*n* = 6), *S. f. affinis* (*n* = 9), and *S.* sp. 1 (*n* = 7) on the 15 craniomandibular variables (log_10_-transformed; Chen et al. 2023). Missing data were imputed from species average.

### Phylogenetic reconstruction and molecular dating.

We used 1 mitochondrial gene (*CytB*) and 6 nuclear gene segments (*Adora3*, *App*, *Atp7*, *Bdnf*, *Brca1*, and *Rag1*) to reconstruct phylogenetic relationships. Forty-nine sequences belonging to 7 individuals were extracted, amplified, and sequenced (Supplementary Data SD3). Total DNA was extracted from muscle or liver tissue using a Qiagen DNeasy blood and tissue kit (Qiagen DNeasy Blood and Tissue Kit, China). Primers and PCR conditions followed He et al. (2019). We downloaded homologous sequences of *Dymecodon pilirostris* True, 1886; *Neurotrichus gibbsii* Baird, 1858; *Urotrichus talpoides* Temminck, 1841; and *Condylura cristata*, Linnaeus, 1758 from GenBank as outgroups (Supplementary Data SD4).

The sequence alignment, selection of substitution scheme, and phylogenetic reconstruction were performed in PhyloSuite (Zhang et al. 2020). The sequences were aligned with MAFFT v7.505 (Katoh and Standley 2013) using the “--auto” strategy and normal alignment mode. We calculated pairwise genetic distances (Kimura-2-parameter, K2P) of the complete *CytB* gene between putative *Scaptonyx* species and outgroups in MEGA X (Kumar et al. 2018). Phylogenetic analyses were conducted using: (i) 1 mitochondrial gene; (ii) concatenated nuclear genes; and (iii) concatenated mitochondrial and nuclear genes. The best partitioning scheme and evolutionary models were selected using PartitionFinder2 v2.1.1 (Lanfear et al. 2017), with the greedy algorithm and AICc criterion. We partitioned the *CytB* gene by codon positions and concatenated alignments by genes for evaluating the best partitioning scheme and evolutionary models following He et al. (2019).

Maximum likelihood phylogenies were inferred using IQ-TREE v2.2.0 (Nguyen et al. 2015) under the model automatically selected by IQ-TREE (“Auto” option in IQ-TREE) for 20,000 ultrafast bootstraps (UFBoot) and the Shimodaira–Hasegawa-like approximate likelihood ratio test (SH-aLRT). Bayesian Inference phylogenies were inferred using MrBayes v3.2.7a (Ronquist et al. 2012) under models selected in the PhyloSuite workflows (2 parallel runs, 5,000,000 generations), with the initial 25% of sampled data discarded as burn-in.

We employed BEAST 2.7.5 (Bouckaert et al. 2019) for divergence dating. The best partitioning scheme and substitution models from the PartitionFinder were used. Following He et al. (2019), we employed the Birth–Death model as tree priors and the relaxed log-normal clock models as the clock model prior; we also constrained monophyly for the *Dymecodon* + *Neurotrichus* + *Scaptonyx* + *Urotrichus* clade and defined the calibration point for the tree root to 41.93 Ma (95% CI = 68.07–34.65 Ma) based on the fossil of *Oreotalpa* (Worley-Georg and Eberle 2006), which was considered to be the oldest relative close to Urotrichini (He et al. 2019). The Star-nosed Mole (*Condylura cristata*) was enforced as the outgroup. We used an estimated time of 25.04 Ma (95% CI: 37.68 to 15.29 Ma) as a secondary calibration for the most recent common ancestor of *Scaptonyx* spp. based on the results of He et al. (2019). We ran the Markov Chain Monte Carlo for 100 million generations with a sampling interval of 10,000 generations. Analyses were repeated 4 times, and the log files and species trees were combined in LogCombiner in BEAST 2.7.5 toolset to improve estimation performance. Posterior distributions and effective sample sizes were calculated using Tracer v1.7. Information from the tree was summarized into a single maximum clade credibility tree in TreeAnnotator with the first 10% of each run discarded as burn-in.

### Distribution delineation based on broadly sampled *CytB* phylogeny.

To determine the distribution range of each species, we reconstructed the *CytB* phylogeny after combining sequences extracted in the present study with 109 sequences downloaded from GenBank, spanning mainland China (Supplementary Data SD5). IQ-TREE was used to build a maximum likelihood tree with 20,000 bootstrap replicates to reconstruct the approximate phylogeny of *Scaptonyx* spp. covering most of the distribution range.

## Results

### Morphological comparison.

**Fig. 2.**
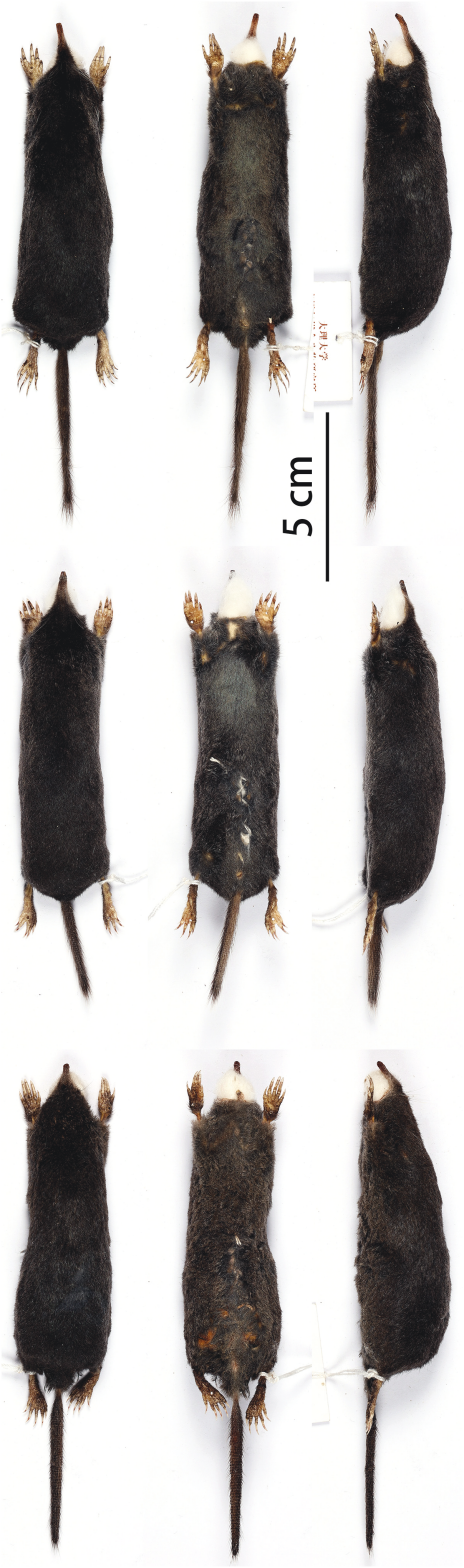
Dorsal, ventral, and lateral views of the skins of *Scaptonyx fusicauda* (KIZ: 042772) (top), *S. affinis* (DLU: BL2307099) (middle), and *S. wangi* sp. nov (KIZ: 042771) (bottom).

**Fig. 3.**
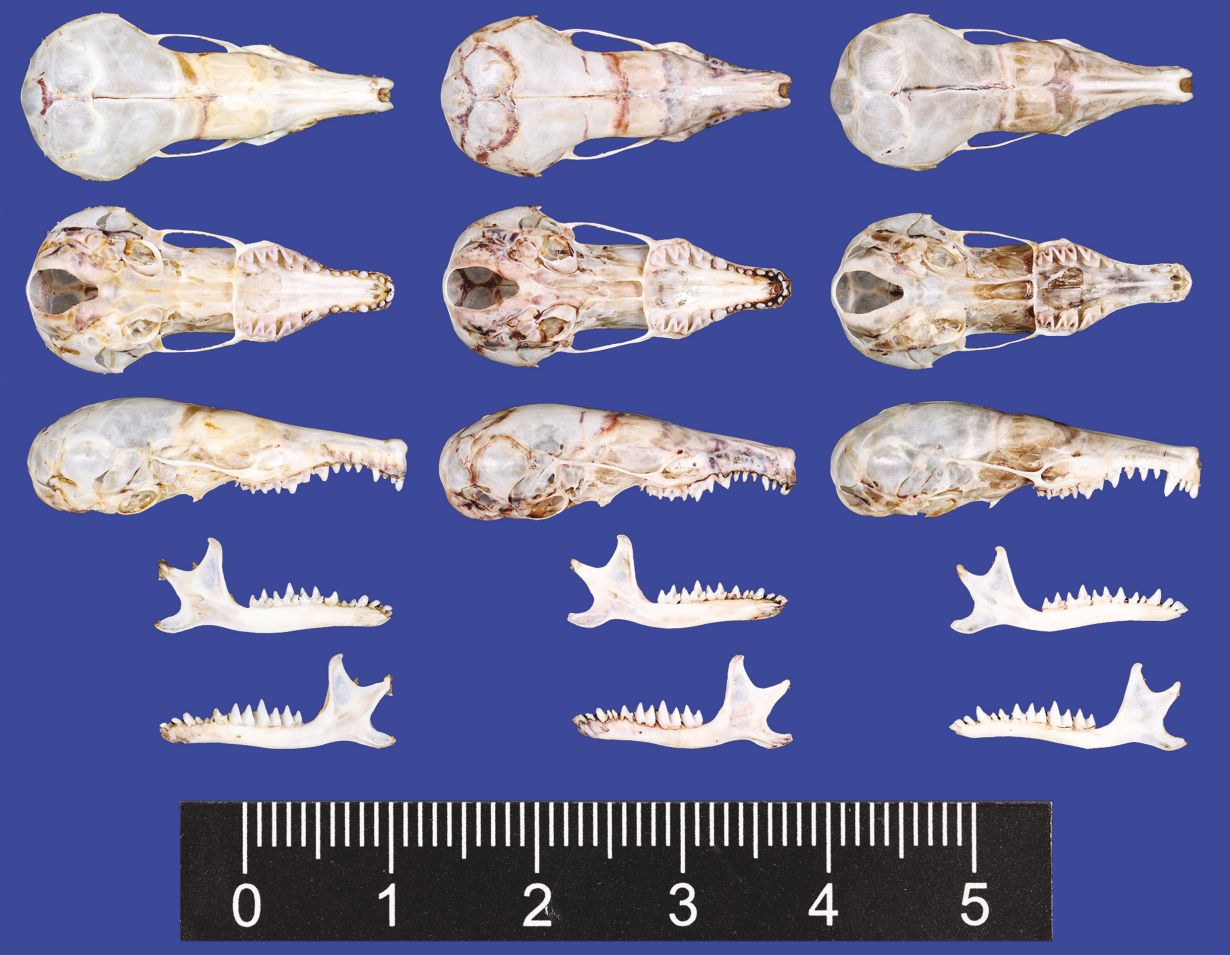
Dorsal, ventral, and lateral views of the skull and lateral views of the mandible of *Scaptonyx fusicauda* (KIZ: 042772) (left), *S. affinis* (DLU: BL2307099) (middle), and *S. wangi* sp. nov (KIZ: 042771) (right). The unit of the scale bar is cm.

**Fig. 4.**
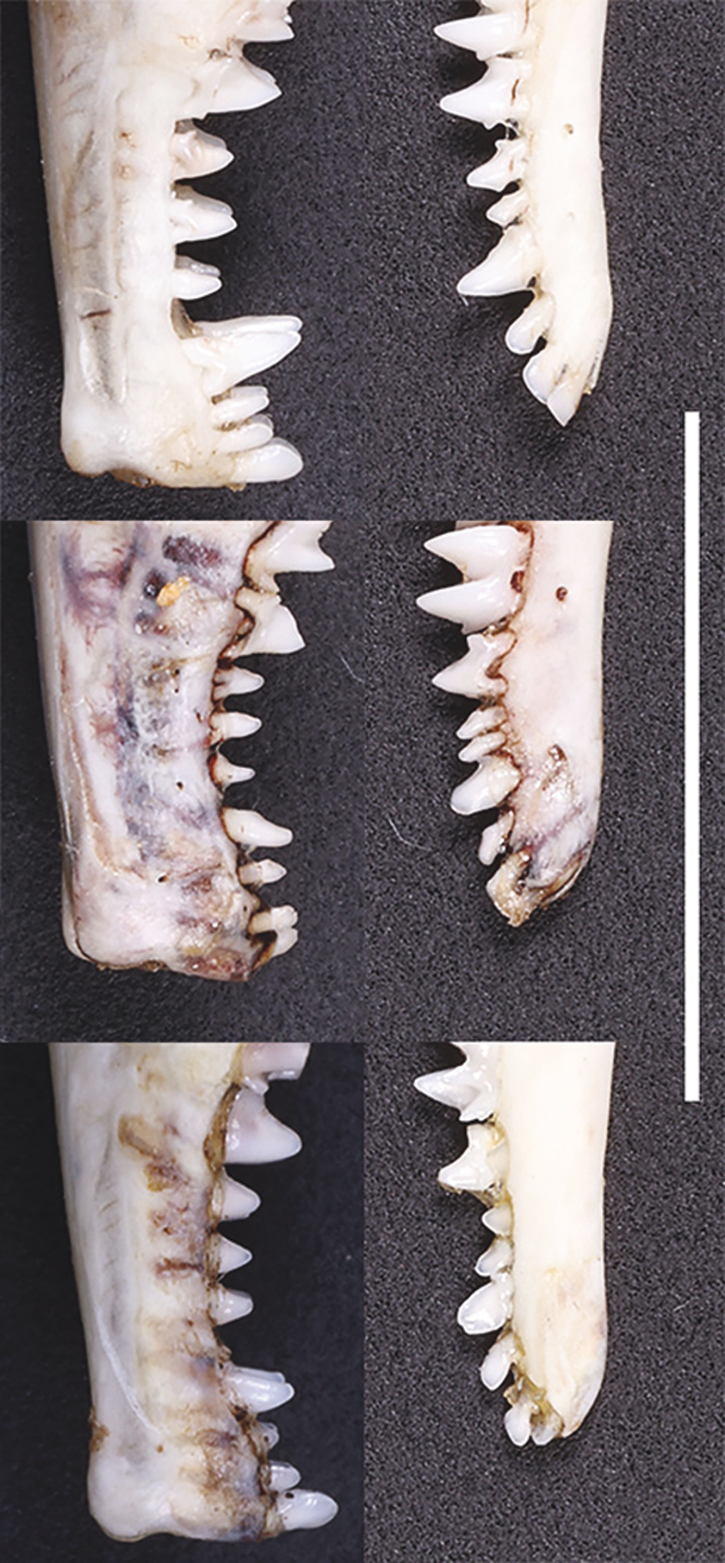
Lateral views of upper and lower incisors, canine, and premolars of *Scaptonyx fusicauda* (left: upper, KIZ: 042772; lower, KIZ: 042773), *S. affinis* (middle: DLU: BL2307099), and *S. wangi* sp. nov (right: KIZ: 042771). The white bar marks a 10 mm length.

Based on visual inspections, *S. fusicauda*, *S. f. affinis*, and *S.* sp. 1 have similar pelage color and limb structure. However, the tail of *S. f. affinis* is distinctively shorter than the others (Fig. 2). The dental formula of examined *Scaptonyx* specimens was I 3/2, C 1/1, P 4/4, M 3/3 (× 2) = 42. From the view of buccal side, *S.* sp. 1 has more robust upper teeth than the other 2 congeners, especially the upper canines (Fig. 3). Moreover, P^1^, P^2^, and P^3^ are about the same size in *S. fusicauda* and *S. f. affinis*, while for *S.* sp.1 P^2^ is larger than P^1^ and P^3^. On the buccal view, P_1_ in *S.* sp.1 is more robust at the base and more pointed towards the tip, making it more triangular than that of *S. fusicauda* and *S. f. affinis* (Fig. 4).

**Fig. 5.**
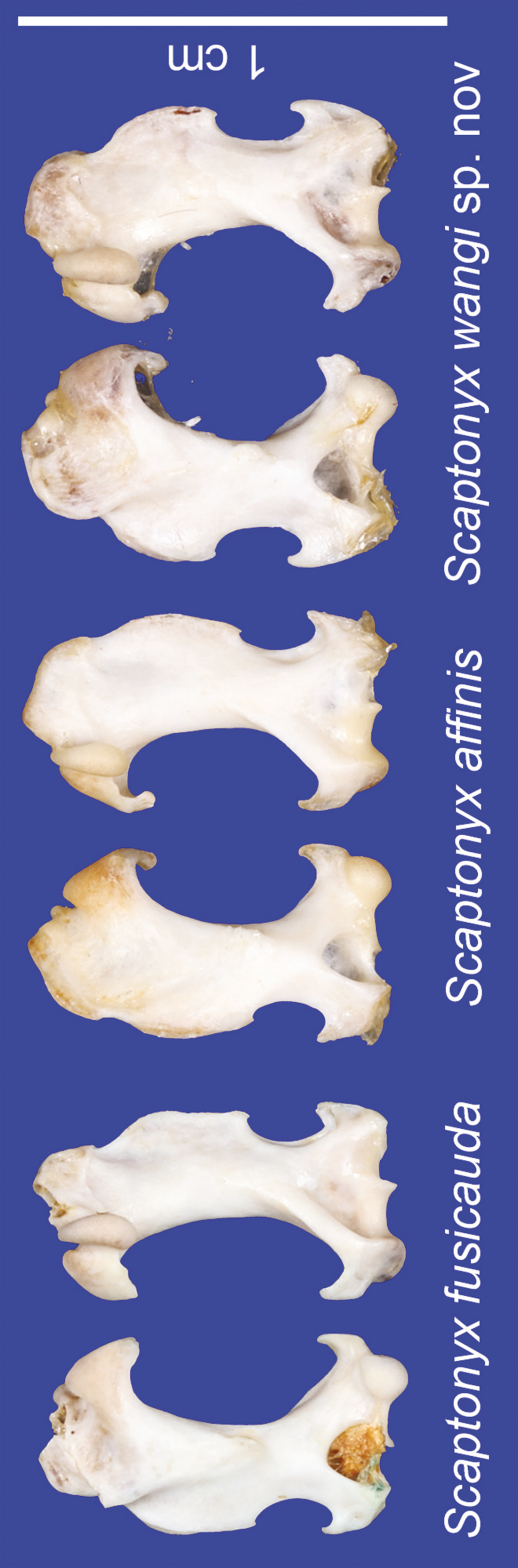
The humeri in anterior (left) and posterior (right) views of *Scaptonyx fusicauda* (KIZ: 042772), *S. affinis* (KIZ: 042775), and *S. wangi* sp. nov (KIZ: 042769).

Humeri among *S. fusicauda*, *S. f. affinis*, and *S.* sp. 1 show contrasting characteristics (Fig. 5). *Scaptonyx fusicauda* has the straightest shaft; distal end of capitulum much stretched out from a distal facet-like process of the medial epicondyle in *S. fusicauda*, while in *S. f. affinis* and *S.* sp. 1 they are at the same level. The humerus of *S. f. affinis* is similar to *S. fusicauda*, but the anterior notch of *S. f. affinis* is shorter than *S. fusicauda*; the pectoralis ridge of *S. f. affinis* is least developed among the putative species. The deltoid process and lateral epicondyle of *S.* sp. 1 exaggeratively curve toward each other, leading to a shorter and more rounded anterior notch. Moreover, the medial epicondyle is shorter than lateral epicondyle in *S. fusicauda* and *S. f. affinis*, and their proximal ends are at the same level. On the contrary, the medial epicondyle is much longer than lateral epicondyle in *S.* sp. 1, resulting in more proximal posterior notch and shorter teres tubercle than the congeners.

Mean values and ranges of external and craniomandibular measurements of examined specimens are provided in Table 1. One-way ANOVA provided strong evidence to support the differences (*P* < 0.05) among *S. fusicauda*, *S. f. affinis*, and *S.* sp. 1, based on 2 external and 13 craniomandibular variables (Table 1). Pairwise differences between *S. fusicauda*, *S. f. affinis*, and *S.* sp. 1 based on the Bonferroni correction tests are shown in Supplementary Data SD6. *Scaptonyx fusicauda affinis* has the shortest tail and a lighter average BW than *S. fusicauda* from the western Sichuan. *Scaptonyx fusicauda* has the largest skull in length, breadth, and height, indicated by strong evidence of differences (*P* < 0.05) in mean values of GLS, PIL, PPL, CB, and CH. *Scaptonyx* sp. 1 possesses a wider IOB than *S. f. affinis* but does not differ from *S. fusicauda*. *Scaptonyx fusicauda* has the longest UTL, while *S.* sp. 1 possesses the shortest P^4^M^3^ and narrowest M^2^M^2^. In the mandibles, *S. fusicauda* has the longest ML, while HCP of *S.* sp. 1 is shorter than the others. *Scaptonyx fisicaudus affinis* exhibits the shortest LTR, whereas LLM for *S.* sp. 1 is shorter than the others.

**Table 1.** Sample size, mean values, standard deviations, and ranges of external and skull measurements of *Scaptonyx* spp. with results of one-way ANOVA for the mean value differences. Variable abbreviations are detailed in the Materials and methods section.

Measurements	Property	*Scaptonyx fusicauda*	*Scaptonyx affinis*	*Scaptonyx waing* sp. nov	*F*	*P*
BW (g)	*n*	6	9	7		
	$\bar{x}$ ± SD	14.8 ± 1.4	12.1 ± 1.6	14.1 ± 2.4	4.196	0.031^a^
	Min.	12.3	10.3	10.3		
	Max.	16	15.1	17.1		
HB (mm)	*n*	6	9	7		
	$\bar{x}$ ± SD	79 ± 5	79 ± 5	81 ± 2	0.522	0.602
	Min.	73	72	79		
	Max.	85	88	83		
TL (mm)	*n*	6	9	7		
	$\bar{x}$ ± SD	47 ± 2	34 ± 2	48 ± 4	59.874	0.000^b^
	Min.	45	31	41		
	Max.	50	39	52		
HF (mm)	*n*	6	9	7		
	$\bar{x}$ ± SD	15 ± 0	14 ± 1	15 ± 1	3.051	0.071
	Min.	14.5	12	13		
	Max.	16	15	17		
GLS (mm)	*n*	4	9	7		
	$\bar{x}$ ± SD	24.97 ± 0.15	23.80 ± 0.29	24.27 ± 0.65	9.915	0.001^b^
	Min.	24.82	23.28	23.23		
	Max.	25.17	24.16	24.8		
PIL (mm)	*n*	6	9	7		
	$\bar{x}$ ± SD	11.13 ± 0.14	10.58 ± 0.19	10.82 ± 0.28	12.428	0.000^b^
	Min.	10.89	10.24	10.44		
	Max.	11.26	10.84	11.13		
PPL (mm)	*n*	4	9	7		
	$\bar{x}$ ± SD	13.85 ± 0.14	13.20 ± 0.11	13.36 ± 0.46	7.049	0.006^b^
	Min.	13.7	13.02	12.69		
	Max.	14.03	13.35	13.89		
CB (mm)	*n*	4	9	7		
	$\bar{x}$ ± SD	11.41 ± 0.22	10.66 ± 0.23	10.59 ± 0.44	9.456	0.002^b^
	Min.	11.26	10.23	9.86		
	Max.	11.73	10.96	11.09		
IOB (mm)	*n*	5	9	7		
	$\bar{x}$ ± SD	5.75 ± 0.11	5.75 ± 0.15	5.98 ± 0.17	5.522	0.013^a^
	Min.	5.61	5.54	5.76		
	Max.	5.9	5.91	6.21		
ZB (mm)	*n*	4	9	7		
	$\bar{x}$ ± SD	8.27 ± 0.06	8.30 ± 0.20	8.14 ± 0.24	1.323	0.292
	Min.	8.22	7.98	7.72		
	Max.	8.36	8.65	8.46		
CH (mm)	*n*	4	9	7		
	$\bar{x}$ ± SD	7.67 ± 0.12	7.18 ± 0.18	7.01 ± 0.34	9.769	0.001^b^
	Min.	7.57	7.05	6.57		
	Max.	7.84	7.58	7.53		
UTL (mm)	*n*	6	9	7		
	$\bar{x}$ ± SD	10.79 ± 0.18	10.13 ± 0.19	10.49 ± 0.27	17.356	0.000^b^
	Min.	10.59	9.86	10.08		
	Max.	11.13	10.4	10.88		
P^4^M^3^ (mm)	*n*	6	9	7		
	$\bar{x}$ ± SD	5.93 ± 0.06	5.84 ± 0.16	5.57 ± 0.20	10.165	0.001^b^
	Min.	5.86	5.63	5.31		
	Max.	6.01	6.11	5.77		
M^2^M^2^ (mm)	*n*	5	9	7		
	$\bar{x}$ ± SD	6.35 ± 0.09	6.34 ± 0.09	6.03 ± 0.22	10.656	0.001^b^
	Min.	6.24	6.22	5.82		
	Max.	6.48	6.47	6.35		
BFM (mm)	*n*	3	9	7		
	$\bar{x}$ ± SD	3.01 ± 0.05	2.89 ± 0.07	2.88 ± 0.15	1.73	0.209
	Min.	2.98	2.76	2.75		
	Max.	3.06	2.95	3.2		
LTR (mm)	*n*	6	9	7		
	$\bar{x}$ ± SD	8.64 ± 0.14	8.09 ± 0.23	8.42 ± 0.17	15.658	0.000^b^
	Min.	8.39	7.73	8.19		
	Max.	8.82	8.38	8.68		
LLM (mm)	*n*	6	9	7		
	$\bar{x}$ ± SD	4.95 ± 0.08	4.85 ± 0.12	4.52 ± 0.23	13.51	0.000^b^
	Min.	4.79	4.67	4.24		
	Max.	5.01	5.03	4.84		
ML (mm)	*n*	6	9	7		
	$\bar{x}$ ± SD	15.68 ± 0.23	15.10 ± 0.20	15.20 ± 0.24	13.125	0.000^b^
	Min.	15.4	14.66	14.99		
	Max.	15.96	15.3	15.58		
HCP (mm)	*n*	6	9	7		
	$\bar{x}$ ± SD	6.01 ± 0.06	5.83 ± 0.16	5.39 ± 0.13	40.199	0.000^b^
	Min.	5.92	5.67	5.2		
	Max.	6.08	6.11	5.64		

^a^ *P* < 0.05.

^b^ *P* < 0.01.

**Fig. 6.**
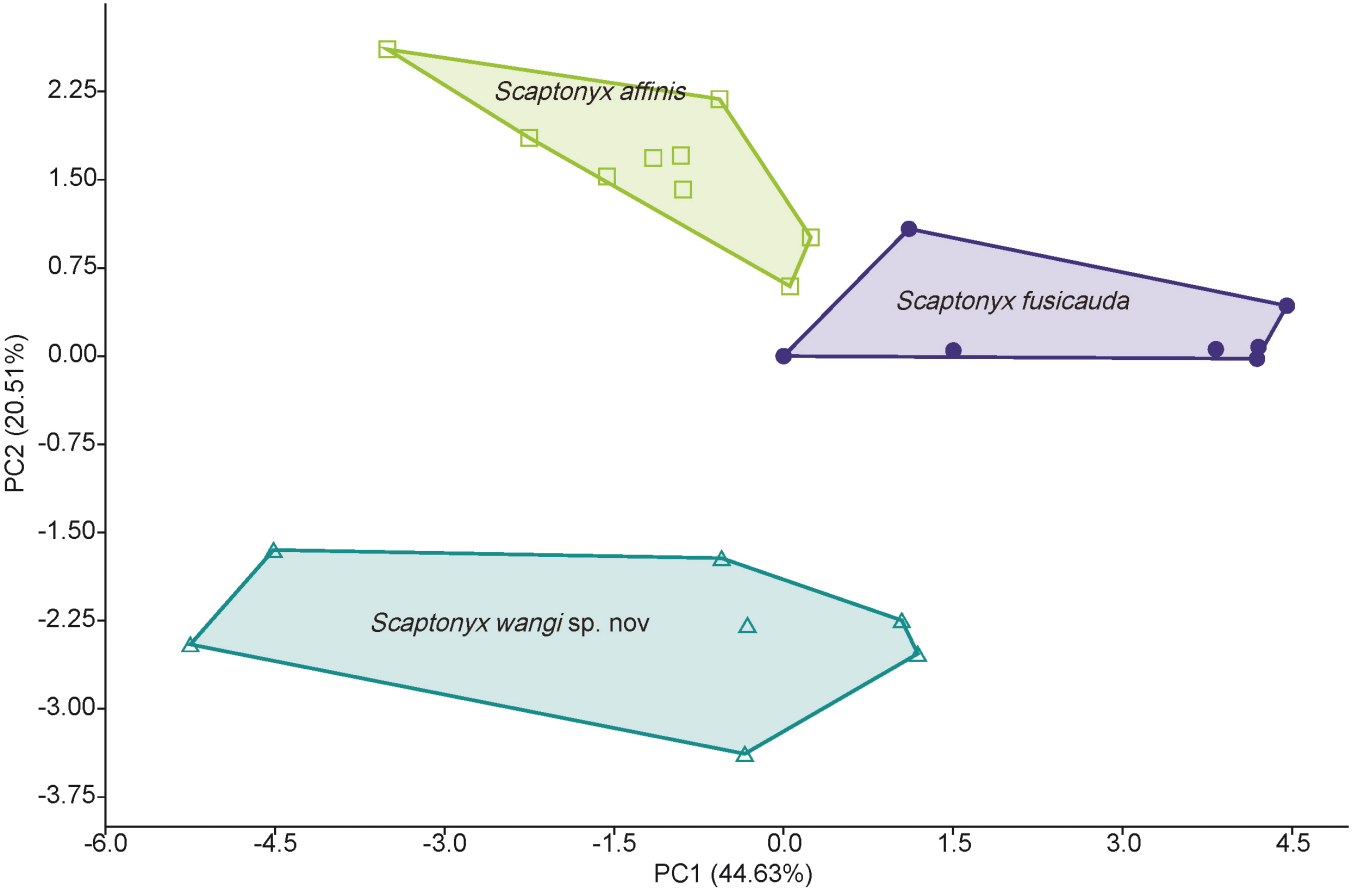
Principal component analyses scatterplot of log_10_-transformed craniomandibular measurements of *Scaptonyx* spp. with variances explained by the first (PC1) and second (PC2) principal components.

The PCA of craniomandibular variables yielded 4 principal components (PCs) with eigenvalues higher than 1, indicating that these components accounted for more variance than accounted by one of the original variables. The first 4 PCs successively explained 44.63%, 20.51%, 10.9%, and 9% (totaling 85.03%) of the variance (Table 2). Loadings on PC1 were positive for all 15 variables and separated specimens mostly based on PIL (0.89), CB (0.89), GLS (0.88), PPL (0.88), and CB (0.82), indicating an association with overall size. PC2 separated the specimens mostly based on HCP (0.74), LLM (0.64), and M^2^M^2^ (0.62), contrasted with IOB (−0.7). PC3 had the highest loading in ZB (0.75), while the loading of BFM (−0.85) mostly represented the variance on PC4. A scatter plot showed that the 3 groups were completely separated from each other on the combination of PC1 and PC2. On PC1, specimens of *S. fusicauda* located in the positive region (Fig. 6), while the other 2 groups were mostly situated in the negative region, indicating that *S. fusicauda* had the largest overall skull size. *Scaptonyx fusicauda affinis* and *S.* sp. 1 were clearly separated on PC2, with *S. f. affinis* on the positive region and *S.* sp.1 on the negative region, indicating that *S.* sp. 1 had a lower coronoid process, shorter lower molar length, narrower width between the second upper molars, and wider interorbital breadth than *S. f. affinis* (Fig. 6).

**Table 2.** Eigenvalue, variance explained, and variable loadings of the first four PC resulted from PCA on the 15 craniomandibular variables. Variable abbreviations are detailed in the Materials and methods section.

Attributes		PC1	PC2	PC3	PC4
Eigenvalue		6.69	3.08	1.64	1.35
Variance explained (%)		44.63	20.51	10.90	8.99
Cumulative % of variance		44.63	65.14	76.04	85.03
Variable loading	GLS	0.88	−0.38	−0.01	0.09
	PIL	0.89	−0.35	−0.18	0.11
	PPL	0.88	−0.23	0.19	0.08
	CB	0.82	0.06	0.39	−0.04
	IOB	0.14	−0.70	0.46	0.32
	ZB	0.39	0.23	0.75	0.33
	CH	0.73	0.30	0.20	−0.40
	UTL	0.77	−0.43	−0.30	0.08
	P^4^M^3^	0.56	0.57	−0.33	0.41
	M^2^M^2^	0.53	0.62	0.21	0.02
	BFM	0.32	0.05	0.19	−0.85
	LTR	0.61	−0.51	−0.34	−0.17
	LLM	0.51	0.64	−0.40	0.14
	ML	0.89	−0.14	−0.19	−0.11
	HCP	0.49	0.74	0.03	0.00

### Phylogenetic relationships and divergence.

The K2P distances based on *CytB* segments were 16.8% between *S. fusicauda* and *S. f. affinis*, 19% between *S. fusicauda* and *S.* sp. 1, and 20.6% between *S. f. affinis* and *S.* sp. 1. The pairwise distances between different populations of *Scaptonyx* exceed the 16.1% distance between *U. talpoides* and the closest *Scaptonyx* member (*S.* sp. 1; Table 3).

**Table 3.** Kimura-2-parameter (K2P) distances based on *CytB* gene.

	*Scaptonyx fusicauda*	*Scaptonyx affinis*	*Scaptonyx wangi* sp. nov	*Condylura cristata*	*Dymecodon pilirostris*	*Neurotrichus gibbsii*
*Scaptonyx affinis*	0.168					
*Scaptonyx wangi* sp. nov	0.190	0.206				
*Condylura cristata*	0.197	0.240	0.221			
*Dymecodon pilirostris*	0.180	0.216	0.189	0.203		
*Neurotrichus gibbsii*	0.208	0.209	0.203	0.224	0.206	
*Urotrichus talpoides*	0.193	0.223	0.161	0.193	0.124	0.185

**Fig. 7.**
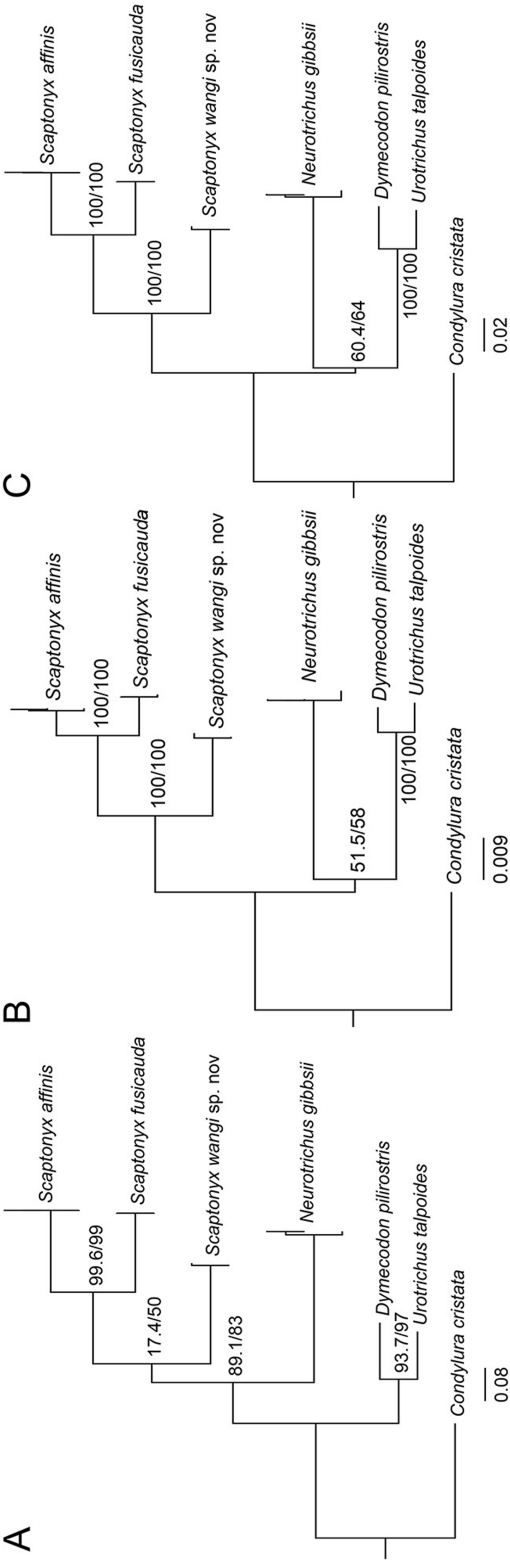
Phylogenetic trees reconstructed from A) *CytB*, B) concatenated nuclear genes, and C) concatenated mitochondrial-nuclear genes using Maximum likelihood analysis. Node labels indicate ultrafast bootstrap support values and SH-like approximate likelihood ratio test support values.

The best partitioning scheme and substitution models were shown in Supplementary Data SD7. The ML and BI phylogenies based on *CytB*, nuclear, and concatenated mitochondrial-nuclear genes showed consistent topologies for the monophyly of genus *Scaptonyx* and 3 clades within *Scaptonyx* (Fig. 7; Supplementary Data SD8). *Scaptonyx fusicauda* (*n* = 2), *S. f. affinis* (*n* = 3), and *S.* sp. 1 (*n* = 2) were respectively monophyletic. Support for the nodes between these 3 clades was robust for phylogenies based on concatenated nuclear genes and concatenated mitochondrial and nuclear genes (Maximum likelihood UFBoot = 100 and SH-aLRT = 100; Bayesian posterior probabilities = 1) (Fig. 7; Supplementary Data SD8).

**Fig. 8.**
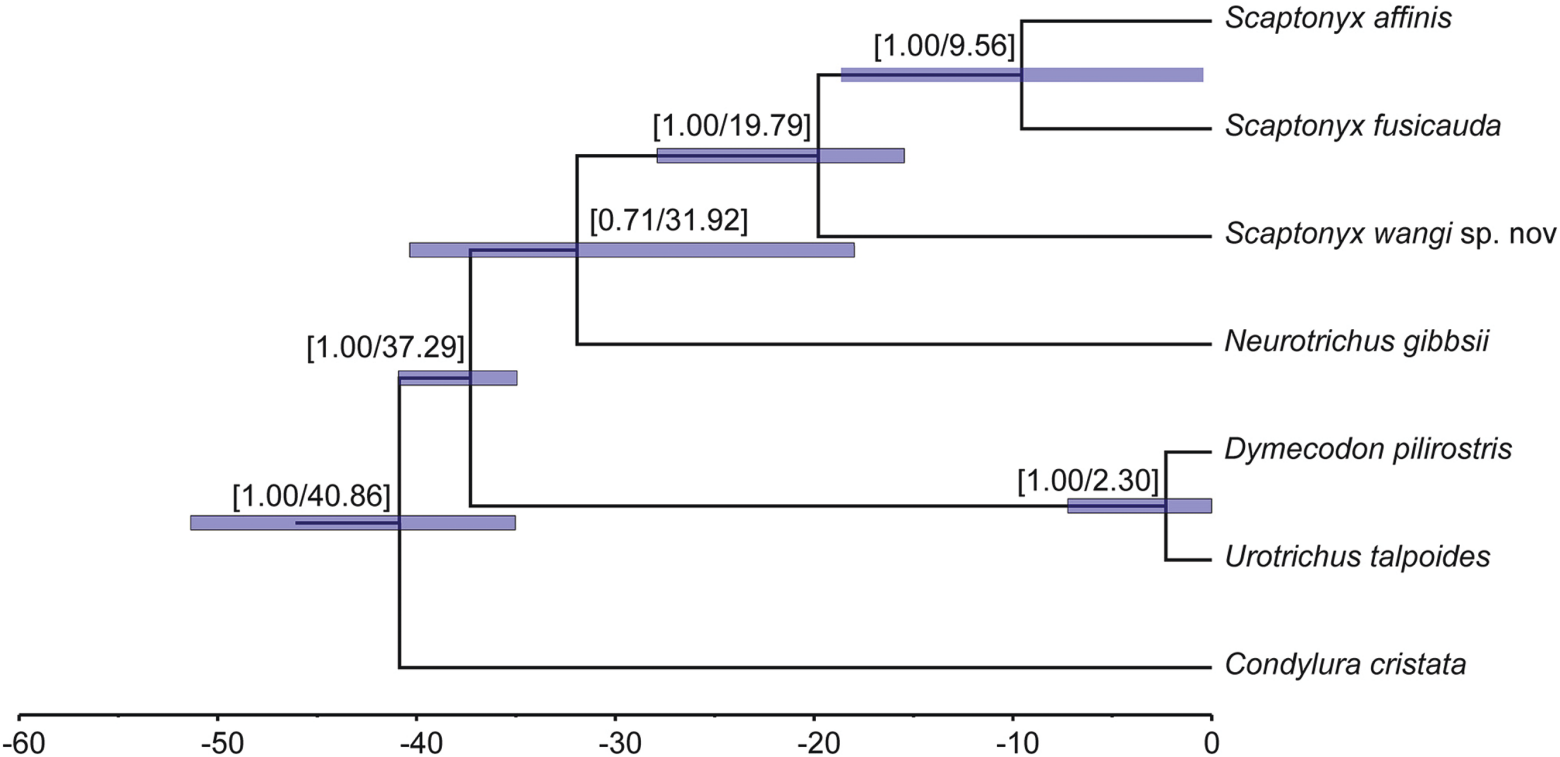
Divergence times estimated based on mitochondrial-nuclear concatenated dataset. Node labels indicate the posterior probabilities and the average divergence times (Ma). Node bars show the estimated 95% CI of the upper and lower boundary of divergence times.

Divergence dating indicated that the ancestors of *Scaptonyx* separated from *Neurotrichus* ca. 31.92 Ma (95% CI: 40.34 to 18 Ma). Within *Scaptonyx*, *S.* sp. 1 diverged from the *S. fusicauda *+ *S. f. affinis* clade ca. 19.79 Ma (95% CI: 27.89 to 15.46 Ma), while *S. f. affinis* and *S. fusicauda* split ca. 9.56 Ma (95% CI: 18.64 to 0.42 Ma; Fig. 8).

### Distribution of the extant species.

The ML tree based on *CytB* sequences, including those downloaded from GenBank, showed that the 3 clades were also geographically delimited: (i) western Sichuan, (ii) western to central China, and (iii) the Gaoligong range (Supplementary Data SD9). Topotypes of *S. fusicauda* clustered with western Sichuan samples, while *S. f. affinis* topotypes clustered with western Yunnan samples, extending to the east side of the Salween River. Individuals from eastern Yunnan and central China were closely related to *S. f. affinis* but showed considerably split lineages. Individuals from the watershed of the Irrawaddy River and the Salween River (Gaoligong range) were grouped with *S.* sp. 1 as a distinct unit. However, samples from the middle (Yaojiaping) and southern (Houqiao) Gaoligong range represented deeply split lineages.

Overall, morphological and phylogenetic evidence supported that *S. f. affinis* should be elevated to a species rank, i.e., *S. affinis*, while *S.* sp. 1 represented an undescribed species. Hence, we comprehensively described the updated taxonomic classification of the genus *Scaptonyx*, along with ecological and distribution information for the extant species.

### Taxonomic accounts.

Family Talpidae Fischer von Waldheim, 1814

Subfamily Talpinae Fischer von Waldheim, 1814

Tribe Urotrichini Dobson, 1883

Genus *Scaptonyx*Milne-Edwards, 1872

#### Type species.

*Scaptonyx fusicauda* Milne-Edwards, 1872

#### Diagnosis and description.

*Scaptonyx* are small-sized moles, HB = 72 to 88 mm, TL = 31 to 52 mm, HF = 13 to 17 mm (Table 1). Dental formula is I 3/2, C 1/1, P 4/4, M 3/3 (× 2) = 42. Pelage is velvet black to grayish black, slightly lighter in the venter than dorsum. The snout is long and narrow. Eyes and the entrance to the external auditory meatus is hidden by pelage. Hands are moderately broadened among the Talpidae family. Hands and feet are scaled, skin velvet black and furry on the lateral side and fresh red and hairless on the medial side. The tail is 40% to 60% of the head-body length. The whole tail is scaly and covered in radially projecting hairs, narrow at the base, thicker in the middle, and rounded at the tip.

Compared with other moles in Asia, *Scaptonyx* is smaller in general body size than the members in tribe Talpini, such as *Euroscaptor, Mogera*, *Parascaptor*, and *Scaptochirus* (Kryštufek and Motokawa 2018). *Scaptonyx* also has slimmer hands than members of subfamily Scalopinae and tribe Talpini, but that are broader than those of Shrew-like Moles (Uropsilinae). *Scaptonyx* can be distinguished from other members in the tribe Urotrichini (including *Dymecodon*, *Neurotrichus*, *Urotrichus*) by the presence of 4 upper and lower premolars and two-rooted upper canine (Van Valen 1967).

#### Distribution

Extant species of *Scaptonyx* occur from central China to northeastern Myanmar and northern Vietnam. Elevational extent ranges between 1,300 and 4,200 m a.s.l.

#### Habitat

In the ground of broad-leaved forests, closed coniferous forest, and rhododendron groves.

*Scaptonyx fusicauda* Milne-Edwards, 1872

*Scaptonyx fusicaudatus* Milne-Edwards, 1872

*Scaptonyx fusicaudus* Milne-Edwards, 1872

#### Holotype

MNHN-ZM-MO-1871-87A, collected by Armand David, deposited in the Muséum National d’Histoire Naturelle, Paris.

#### Type locality.

In Milne-Edwards (1872), “confins du Kokonoor et du Sé-tschouan” (= borders of Qinghai and Sichuan). We refined the type locality as within the vicinity of the main source of the Minjiang River (ca. 33.03° N, 103.71° E) on Minshan Mountain, in the boundary between Songpan and Jiuzhaigou Counties, Sichuan, China.

#### Specimens examined.

Skins and skulls of 2 specimens (042772, 042773) deposited in Kunming Institute of Zoology, Chinese Academy of Science and 5 specimens (181729, 181578, 181579, 06383, 王朗-06-06-D2-01) in the Sichuan Academy of Forestry, Sichuan, China (Appendix I).

#### Diagnosis and description.

Head-body length 73 to 85 mm, TL 45 to 50 mm, HF 14 to 16 mm; Table 1. The pelage is uniformly grayish black. The tail is velvet black in dorsum and blackish brown in venter, slightly longer than 50% of the head-body length. Although external and craniomandibular measurements largely overlap between the specimens in the genus, the skull of *S. fusicauda* from the Minshan area is longer, wider, and higher than those specimens from the Baima Mountain and the Gaoligong Mountains, indicated by the measurements including GLS, PIL, PPL, CB, CH, ML, and HCB (Table 1). Viewed from buccal aspect, the first incisor is quite spatulate, larger than twice the width and height of I^2^ and I^3^; the upper canine twice the height and about the same width of P^1^; the P^1^, P^2^, and P^3^ are about the same width and height, half the height and one third the width of P^4^; the P_1_ is enlarged and oval-shaped, one and a half the height and twice the width of the lower canine, and about the same width and height of P_4_.

#### Remarks

Because the type locality was ambiguous and the holotype skull was damaged (Milne-Edwards 1872), we restricted the type locality from thousands of kilometers to a specific mountain area and obtained topotypes for taxonomic reevaluation. Thomas (1912) noted that the dental formula in Milne-Edwards (1872) was incorrect and provided a correction (see description for the genus). This taxon has several specific names with different Latinized ending, including “*fusicauda*” (David 1871), “*fusicaudatus*” (Milne-Edwards 1872), and “*fusicaudus*” (Ellerman and Morrison-Scott 1951). Based on Article 32.5.1 and 33.3.1 in ICZN (1999), all these specific names should be considered correct. We recommend *S. fusicauda* here owing to the Principle of Priority. Suggested common name: Western Sichuan long-tailed mole; 川西长尾鼹.

#### Distribution

*Scaptonyx fusicauda* is endemic to China and distributed in the plateaus and mountainous areas surrounding the Sichuan Basin to the west. Its distribution ranges in elevation from 1,700 m to 3,400 m a.s.l.

#### Habitat

Inhabits broad-leaved forest and closed coniferous forest with ample fallen woods and thick humus.

*Scaptonyx affinis* Thomas, 1912

*Scaptonyx fusicauda affinis* Thomas, 1912

*Scaptonyx fusicaudatus affinis* Thomas, 1912

*Scaptonyx fusicaudus affinis* Thomas, 1912

#### Holotype

NHM 1912.12.3.18.1, collected 22 June 1911. Specimen is currently deposited at the Natural History Museum, London, UK.

#### Type locality

“12 miles S.E. of A-tun-tsi. 13,500.” A-tun-tsi is spelled A-dun-zi (阿墩子) in modern Chinese Pinyin. It is the ancient name for Deqin County in northwest Yunnan, China. Twelve miles (ca. 20 km) southeast of Deqin is now known as Quzonggong.

#### Specimens examined.

Skins and skulls of 3 specimens (042774, 042775, 042776) deposited in the Kunming Institute of Zoology, Chinese Academy of Science and 6 specimens (BL2307032, BL2307033, BL2307034, BL2307052, BL2307099, BL2307163) deposited in the Dali University, Yunnan, China (Appendix I).

#### Diagnosis and description.

Slightly smaller than *S. fusicauda*, based on its lighter BW and shorter head-body length (HB: 72 to 88 mm, TL: 31 to 39 mm, HF: 12 to 15 mm). The tail is ca. 40% of head-body length. The pelage is similar to that of *S. fusicauda* but the tail is velvet black in dorsum and flesh red in venter, which makes it contrastingly bi-colored. In the buccal aspect, the I^1^ and I^2^ are about the same size and spatulate, while I^3^ is shorter and sharp; upper canine is about one and a half the width and height of I^3^; P^1^, P^2^, and P^3^ are subequal, although some individuals have a slightly larger P^3^; the P^4^ crown is enlarged, almost forming a regular triangle, with the same height and twice the breadth of upper canine. The oval-shaped P_1_ is 4 times the width and one and a half times the height of P_2_ and P_3_, and it is about the same height as P_4_.

#### Remarks

The subspecies *S. f. affinis* described by Thomas (1912) was widely recognized by taxonomists (Anthony 1941; Ellerman and Morrison-Scott 1951; Hill 1962; Wang 2003; Wilson and Reeder 2005; Smith et al. 2010; Kryštufek and Motokawa 2018). Our results indicate that *S. affinis* is a distinct species. Morphologically, this species can be distinguished from *S. fusicauda* by its smaller body size, distinctively shorter and contrastingly lighter color in venter of the tail. Suggested common name: Deqin long-tailed mole; 德钦长尾鼹.

#### Distribution

*Scaptonyx affinis* is primarily distributed from southwest to central China. A single specimen was recorded in northern Vietnam (Mt. Tay Con Linh II) (Lunde et al. 2003). In Yunnan, it occurs in the mountains of Nushan, Yunling, and Shaluli ranges (Zhang 1997) and hills in the southern and eastern parts, limited to the east side of Nujiang (Salween River). Out of Yunnan, its distribution extends to central China, including the Dalou, Bashan, and Qingling mountains. The distribution of this species spans an elevational extent between 1,300 m and 4,200 m a.s.l.

#### Habitats

*Scaptonyx affinis* is found in evergreen broad-leaved forests, closed coniferous forest, and rhododendron groves.

*Scaptonyx wangi* sp. nov. Song and Jiang

#### Holotype

KIZ 042771, adult male, collected on 26 June 2022 by Da-Zhou Peng. Dried skin, cleaned skull, and alcohol-preserved carcass deposited in Kunming Institute of Zoology, Chinese Academy of Science.

#### Type locality.

Closed coniferous forest outside the east entrance of the Dulongjiang Tunnel (27.806° N, 98.459° E, 3 092 m.a.s.l.) on the road from Gongshan County to Dulongjiang Township, Nujiang Lisu Autonomous Prefecture, Yunnan Province, China.

#### Paratypes

Skins and skulls of 6 specimens (042765, 042766, 042767, 042768, 042769, 042770) were deposited in the Kunming Institute of Zoology, Chinese Academy of Science (Appendix I).

#### Diagnosis

Head-body length 79 to 83 mm, TL 41 to 52 mm. Tail longer than 50% of head-body length. From a lateral perspective, the anterior tip of the nasal bone does not extend beyond the anterior facet of I^1^. It is distinguishable in the genus by the remarkably enlarged upper canine and the triangular lower first premolar (Fig. 4). P^2^ is higher than P^1^ and P^3^.

#### Description

A small-sized mole (BW: 14.1 ± 2.4 [$\bar{x}$ ± SD] g, range 10.3 to 17.1 g; HB: 81 ± 2 mm, range 79 to 83 mm; TL: 48 ± 4, range 41 to 52 mm; HF: 15 ± 1 mm, range 13 to 17 mm; Table 1). The pelage is velvet black, darker on the dorsum, and slightly lighter on the venter. The tail is longer than 50% of the head-body and covered in sparse black hairs. The skin of the tail is similar to *S. fusicauda*, velvet black in dorsum and blackish brown in venter. Four of the 7 individuals examined have long, tufted hairs (ca. 1 cm long) at the end of the tail.

The dorsal view of the skull is narrowly triangular. In the lateral perspective, the anterior tip of the nasal bones does not project beyond I^1^. The braincase is narrow (CB: 9.86 to 11.09 mm) and low (CH: 6.57 to 7.53 mm) (Table 1). The posterior margin of the palate is straight or slightly convex posteriorly. In the buccal aspect, I^1^ is more spatulate and higher and wider than I^2^ and I^3^. The upper canine is more than 3 times the width and twice the height of I^3^ and P^1^. P^1^ and P^3^ are subequally small, about three-fourths the height of P^2^. P^4^ is twice the width and height of P^3^.

The body of the mandible is narrow and long. The coronoid process is high and straight, with a narrow tip. In the buccal aspect, the 2 lower incisors are spatulate and subequal, followed by an oval-shaped canine of the same size; enlarged P_1_ is triangular-shaped with a sharp tip; P_3_ is twice the breadth and one and a half the height of P_2_, P_4_ is quadruple the breadth and more than twice the height of P_2_.

#### Etymology

The species name is in honor of Professor Ying-Xiang Wang. He joined the mammal research group at the Kunming Institute of Zoology, Chinese Academy of Sciences in 1962 and led the group for decades until retirement (Jiang 2016). He has made significant contributions to mammalogy in China by contributing to extensive research on the taxonomy, phylogeny, zoogeography, and conservation of mammals. The legacy he left to the students is also reflected in his passionate enthusiasm for challenging field investigations. Suggested common name: Gaoligong Long-tailed Mole; 高黎贡长尾鼹.

#### Comparison

*Scaptonyx wangi* is similar to *S. fusicauda* in external appearance, including pelage color, head-body shape, and limb structure, but differs from *S. affinis* in having a longer tail. The new species is distinctive from other *Scaptonyx* in the prominently developed upper canine teeth, the triangular P_1_ from buccal aspect, and a P_3_ higher than P_2_. Moreover, the lateral view of the anterior nasal tip in *S. wangi* ends posterior to I^1^. In contrast, the tip of the nasal extends anterior to I^1^ in *S. fusicauda* and *S. affinis*. The humerus of *S. wangi* can be distinguished from that of its congeners by the more closed deltoid process and lateral epicondyle, longer medial epicondyle than lateral epicondyle, more proximal posterior notch, and shorter teres tubercle (Fig. 5).

*Scaptonyx wangi* also can be distinguished from *S. fusicauda* by the following: (i) most of the craniomandibular variables of the new species are slightly smaller than those of *S. fusicauda*, e.g., GLS, PIL, PPL, CB, CH, LLM, ML, and HCP (Table 1); (ii) the dental measurements also are slightly smaller in *S. wangi* than in *S. fusicauda*, e.g., P^4^M^3^, M^2^M^2^, and LLM (Table 1); (iii) in *S. wangi,* P^2^ is higher than P^1^ and P^3^, while in *S. fusicauda* P^1^, P^2^, and P^3^ are about the same height (Fig. 4); (iv) the humerus in *S. wangi* differs from that of *S. fusicauda* by the less stretched distal end of capitulum (Fig. 5).

*Scaptonyx wangi* can be differentiated from *S. affinis* by a longer tail (48 ± 4 mm vs. 34 ± 2 mm). The dorsal and ventral skin colors of *S. wangi*’s tail were close, while in *S. affinis* the ventral skin color of the tail is much lighter than the dorsum. The 2 species also have several craniomandibular differences. *Scaptonyx wangi* generally has a narrower rostrum and palate than *S. affinis*, indicated by the smaller ZB and M^2^M^2^ measurements (Table 1). *Scaptonyx wangi* can also be distinguished from *S. affinis* by the enlarged upper canine and P_1_ (Fig. 4). In *S. wangi*, the upper canine is 3 times the width and more than twice the height of P^1^; P_1_ is more than 3 times the height and width of P_2_. In *S. affinis*, the upper canine is less than twice the height and width of P^1^; P_1_ is less than twice the height of P_2_. Moreover, in *S. wangi* I^1^ is much higher and more spatulate than in *S. affinis* (Fig. 4); crown of P^2^ is higher than P^1^ and P^3^, while in *S. affinis* they are approximately the same height. Incisors, canines, and premolars in the lower jaw are dorsoventrally higher and anteroposteriorly wider in *S. wangi* than *S. affinis* (Fig. 4). The wider teeth in the lower jaw also lead to a longer LTR (8.42 ± 0.17 mm vs. 8.09 ± 0.23 mm) despite the LLM (4.52 ± 0.23 mm vs. 4.85 ± 0.12 mm) is shorter in the former (Table 1; Supplementary Data SD6). P_2_ is much lower than P_3_ in *S. wangi*, but they are about the same height in *S. affinis*.

#### Distribution

*Scaptonyx wangi*’s distribution spans the Gaoligong Mountains, defined by the watershed of the Irrawaddy River and the Nu (= Salween) River, encompassing northeastern Myanmar and western Yunnan, at elevations ranging from 2,600 to 4,000 m a.s.l.

#### Habitats

*Scaptonyx wangi* generally inhabits mixed broad-leaved forests, closed coniferous forests, and rhododendron groves, with thick humus forming the ground surface.

## Discussion

For more than a century, the type locality of the genus *Scaptonyx* and its type species *S. fusicauda* was considered to be at the border of Qinghai and Sichuan (Ellerman and Morrison-Scott 1951; Kryštufek and Motokawa 2018; Wang et al. 2023) because “Kokonoor” is the Mongolian name for the Qinghai Lake and was used to refer to Qinghai Province (David 1871, 1875). Nevertheless, this ambiguous description left taxonomists a puzzle to be resolved, and the grassland surrounding the lake is not suitable for *Scaptonyx* spp. which only inhabits forests.

The type specimen of *S. fusicauda* was collected and sent to Paris by Armand David (Milne-Edwards 1872). At the end of 1869, after spending 9 months in Mouping (presently Baoxing), Armand David took a quick excursion to “Kokonoor” from Chengdu (David 1871). He first reached Longanfou (presently Pingwu) in the northeast, then marched northwest shortly. He believed this journey finally took him to the border of “Kokonoor,” where he obtained the holotype of *S. fusicauda* (David 1871). Maps in David (1875) and Bishop (1990) showed that Armand David’s journey after Longanfou ended near the source of a north-south flowing Minjiang River, which runs through Songpan and passes Chengdu from the west. Additionally, in his diary, Armand David believed that “Sungpan (= Songpan)” was bordering “the famous Koko Nor,” which he showed a great interest in visiting (Fox 1949). He also met the Si-Fan people in the place he believed to be the easternmost part of “Kokonoor” (David 1875). These people were known to live in Songpan (Wilson 2011).

All these narratives pointed to the notion that Armand David had been to Songpan and the adjacent areas (including Jiuzhaigou) and obtained the type specimen of *S. fusicauda* there. These areas include Minshan Mountain and, more likely, the west slope of it and near to the source of Minjiang River, which is very close to the border of “Kokonoor” in Armand David’s map (Fig. 1; David 1875). Nevertheless, Armand David probably did not reach “Kokonoor,” nor the border of Qinghai and Sichuan in the late Qing dynasty. This is because he seemed to have misconceived this border, which was inconsistent with the Chinese administrative divisions of that era (Fu et al. 2017). After reviewing relevant literature, we revised the type locality of *S. fusicauda* and collected 2 specimens in the north of the Minjiang River source that could adequately serve as the topotypes of *S. fusicauda* in taxonomic comparison between those from various locations.

Although we found that *S. wangi* from the Gaoligong Mountains was more distant from other *Scaptonyx* species than from *U. talpoides* based on the *CytB* gene, different gene combinations and morphological characteristics still suggested that *S. wangi* belongs to the genus *Scaptonyx*. Results also supported the divergent pattern of the 3 *Scaptonyx* species recognized in the present study, which correspond to the 3 main clades reported by He et al. (2019): *S. wangi* in “Clade I”; *S. fusicauda* in “Clade II”; and *S. affinis* in “Clade III.” These findings provide strong support for the phylogeny-based taxonomic hypotheses of He et al. (2019) by including topotypes of each species in the phylogeny. Based on the species distribution determined by the broadly sampled *CytB* phylogeny, we confirm that the western Sichuan samples belong to a monophyletic clade of *S. fusicauda* topotypes. The distribution of this species is restricted to the plateaus and mountainous areas in western Sichuan. The recently released complete mitochondrial genome (Wang et al. 2023) likely belongs to *S. fusicauda* from the Minshan area. We hypothesize that the Jinsha River and Yalong River served as main barriers for westward and southward dispersion of *S. fusicauda*.

Our morphological comparison demonstrated that the subspecies described by Thomas (1912) showed distinctive external and skull characteristics compared to *S. fusicauda* from western Sichuan and *S. wangi*. Moreover, the phylogenies based on mitochondrial and nuclear genes supported *S. affinis* forming a monophyletic lineage, split from *S. fusicauda* during the late Miocene (9.76 Ma). These results support that this subspecies should be recognized as a distinct species. Based on existing molecular species delimitation (He et al. 2019) and our results, *S. affinis* should include the populations in western Yunnan and be limited to the east of the Salween River. Samples from central China and middle and eastern Yunnan, respectively, comprise several sister clades close to *S. affinis* (He et al. 2019). The specimen from northern Vietnam morphologically corresponds to *S. affinis* (Lunde et al. 2003). Hence, we temporarily assigned populations from these localities under *S. affinis* and defined its distribution extent. According to a recent molecular study, the taxonomic status of populations in southern to central China (e.g., Dalou, Bashan, and Qingling Mountains) may represent undescribed taxa (He et al. 2019), which implies that *Scaptonyx* contains additional species. Further taxonomic work integrating morphological comparisons and phylogenetic analysis is needed.

We observed sufficient morphological distinctions and identified a deep split in the early to middle Miocene (19.79 Ma) of *S. wangi* from other congeners to support its taxonomic validity. This result is consistent with previous phylogeographic and demographic analyses (He et al. 2017, 2019). The 3 parallel rivers (from west to east—Salween, Mekong, and Jinsha) in southwest China have been hypothesized to be geographical barriers for various animal taxa (Wan et al. 2021). It has been shown that the Gaoligong Mountains per se harbor high mammal, bird, and herp diversity and act as an eastern limit of Indo-Burma mammals (Li et al. 2024; Wang et al. 2024; Wu et al. 2024). Early geological studies suggested that a rapid incision of the Salween River happened in the early to middle Pleistocene (~2 Ma; He et al. 1992), which was younger than the Mekong River (~17 Ma) and at about the same time as the modern Jinsha River (1.5 to 3.4 Ma; Cheng et al. 2001). Interestingly, our results suggest that divergence between species and lineages from opposite sides of the Salween River is higher than those from opposite sides of the Mekong River, which also has been reported in He et al. (2019). Moreover, previous biogeographical analyses revealed a high divergence of animal fauna on opposite sides of the Salween River, e.g., Asian shrew-like moles (Wan et al. 2018), flying squirrels (Li et al. 2019, 2021), and white-bellied rats (Ge et al. 2018). These studies indicate that the Salween Valley might have acted as a barrier prior to the formation of the river. Recent dating of apatite found in Salween indicated that this valley experienced 2 phase erosions at 11 Ma and 8 Ma (Yang et al. 2016; Wang et al. 2018), which was ~ 5 Ma older than the apatite date of 3 Ma from Mekong Valley (Replumaz et al. 2020). However, more geological evidence is required towards a better understanding to the formation history of the Salween Valley (Replumaz et al. 2020), preventing us from further discussing its barrier effect on animal fauna diversification and dispersal.

Extant *Scaptonyx* spp. share similar straightened shafts of humeri with the other members in the tribe Urotrichini (Sánchez-Villagra et al. 2004; Piras et al. 2012), which are less specialized for burrowing than those in tribes Scalopini and Talpini but contrasted with shrew-like moles. Nevertheless, the stronger humerus of *S. wangi* may indicate that this species is a better excavator than *S. fusicauda* and *S. affinis* (Sansalone et al. 2018). It is not clear whether this is associated with differential life history and environmental factors resulting from regional disparity. A comprehensive inter-specific comparison among the Talpidae species may provide better insight into the functional adaption of these phenotypic variations.

## Supplementary data

Supplementary data are available at *Journal of Mammalogy* online.

**Supplementary Data SD1**. External and craniomandibular measurements of examined specimens.

**Supplementary Data SD2**. Craniomandibular variables were measured in the present study for morphological comparison. Variable abbreviations are detailed in the Materials and methods section.

**Supplementary Data SD3**. Sequences extracted in the present study for phylogeny reconstructions.

**Supplementary Data SD4**. Species and sequences used as outgroups for divergence dating analyses.

**Supplementary Data SD5**. The list of *CytB* Sequences of Scaptonyx spp. downloaded from GenBank.

**Supplementary Data SD6**. Results of Post Hoc test based on Bonferroni correction for external and craniomandibular variables.

**Supplementary Data SD7**. Partition schemes and evolutionary models are used for concatenated mitochondrial and nuclear phylogenetic analyses.

**Supplementary Data SD8**. Phylogenetic trees were reconstructed from A) *CytB*, B) concatenated nuclear, and C) concatenated mitochondrial-nuclear genes using Bayesian Inference analysis. Node labels are Bayesian posterior probabilities. Scale bars represent substitutions per site.

**Supplementary Data SD9**. Maximum likelihood tree based on the *CytB* gene combining newly extracted sequences and sequences downloaded from GenBank. Highlighted sequences mark the sequence extracted in the present study. Node labels indicate bootstrap values (20,000 replicates) and SH-like approximate likelihood ratio test supports (SH-aLRT).

gyae142_suppl_Supplementary_Datas_SD1

gyae142_suppl_Supplementary_Datas_SD2

gyae142_suppl_Supplementary_Datas_SD3

gyae142_suppl_Supplementary_Datas_SD4

gyae142_suppl_Supplementary_Datas_SD5

gyae142_suppl_Supplementary_Datas_SD6

gyae142_suppl_Supplementary_Datas_SD7

gyae142_suppl_Supplementary_Datas_SD8

gyae142_suppl_Supplementary_Datas_SD9

## Data Availability

All new DNA sequences obtained in this study were deposited in GenBank (https://www.ncbi.nlm.nih.gov/, accession numbers PP354078–PP354084 and PP458486–PP458527). Original contributions presented in the study are included in the article/Supplementary material and deposited in ScienceDB (https://www.scidb.cn/en).

## Notes

Version of Record, first published online 15 March, 2025, with fixed content and layout in compliance with Art. 8.1.3.2 ICZN.

## Appendix I

Specimens examined for this study. Institute abbreviations: KIZ = Kunming Institute of Zoology, Chinese Academy of Science; SAF = Sichuan Academy of Forestry, Sichuan, China; DLU = Dali University, Yunnan, China.

*Scaptonyx fusicauda* (*n* = 7).—KIZ: 042772 (♀), 042773 (♂), collected from Guanmenzi (关门子, 33.133 N, 103.728 E [datum: WGS84], 2 700 m a.s.l.), Zhangzha Township, Jiuzhaigou County, Aba Tibetan and Qiang Autonomous Prefecture, Sichuan Province, China; SAF: 181729 (♂, 32.977 N, 104.081 E [datum: WGS84], 2 600 m a.s.l.), 181578 (♀, 32.996 E, 104.019 N [datum: WGS84], 2 880 m a.s.l.), 181579 (♂, 32.996 N, 104.019 E [datum: WGS84], 2 880 m a.s.l.), 06383 (♂, coordinates unavailable), 王朗-06-06-D2-01 (sex unavailable, coordinates unavailable), collected from Wanglang Nature Reserve, Pingwu County, Sichuan Province, China. Specimens were prepared as dried skins and cleaned skulls.

*Scaptonyx affinis* (*n* = 9).—KIZ: 042774 (♀, 28.342 N, 99.034 E [datum: WGS84], 3 992 m a.s.l.), 042775 (♀, 28.342 N, 99.034 E [datum: WGS84], 3 992 m a.s.l.), 042776 (♀, 28.339 N, 98.985 E [datum: WGS84], 4 200 m a.s.l.), collected from Quzonggong (曲宗贡), Baima Mountain, Deqin County, Yunnan Province, China; DLU: BL2307032 (♀), BL2307033 (♂), BL2307034 (♂) (28.307 N, 99.150 E [datum: WGS84], 3 677 m a.s.l.), BL2307052 (♂), BL2307099 (♀), BL2307163 (♂) (28.307 N, 99.129 E [datum: WGS84], 3 577 m a.s.l.), collected 10 km southeast of Quzonggong, Baima Mountain, Deqin County, Yunnan Province, China. Specimens were prepared as dried skins, cleaned skulls, and alcohol-preserved carcasses.

*Scaptonyx wangi* sp. nov (*n* = 7).—KIZ: 042771 (♂, holotype, 27.806 E, 98.459 N [datum: WGS84], 3 092 m a.s.l.) collected in the dark coniferous forest outside the east entrance of the Dulongjiang Tunnel on the road from Gongshan County to Dulongjiang Township, Nujiang Lisu Autonomous Prefecture, Yunnan Province, China, 042765 (♂, 28.107 N, 98.189 E [datum: WGS84], 3 826 m a.s.l.), collected from Baimalaka of Dulongjiang Township, 042766 (♀, 27.213 N, 98.702 E [datum: WGS84], 3 569 m a.s.l.), collected from Yaping mountain pass of the Shiyueliang Township, 042767 (♀, 26.057 N, 98.681 E [datum: WGS84], 3 623 m a.s.l.), 042768 (♂, 26.064 N, 98.686 E [datum: WGS84], 3 410 m a.s.l.), collected from Tingminghu in Pianma Township, 042769 (♂, 27.964 N, 98.521 E [datum: WGS84], 3 387 m a.s.l.), collected from Jiagetong of Bingzhongluo Township, 042770 (sex unavailable, 27.769 N, 98.447 E [datum: WGS84], 3 493 m a.s.l.), collected from the vicinity of the Dulongjiang Tunnel. Specimens were prepared as dried skins, cleaned skulls, and alcohol-preserved carcasses.
