# Supplementary figures and images for: Taxonomic revision of the Long-tailed Mole (Talpidae: Scaptonyx) with description of a new species from the Gaoligong Mountains
Source: J Mammal. 2025 Mar 15;106(3):782–97. doi: 10.1093/jmammal/gyae142 (PMC12159532; doi:10.1093/jmammal/gyae142)

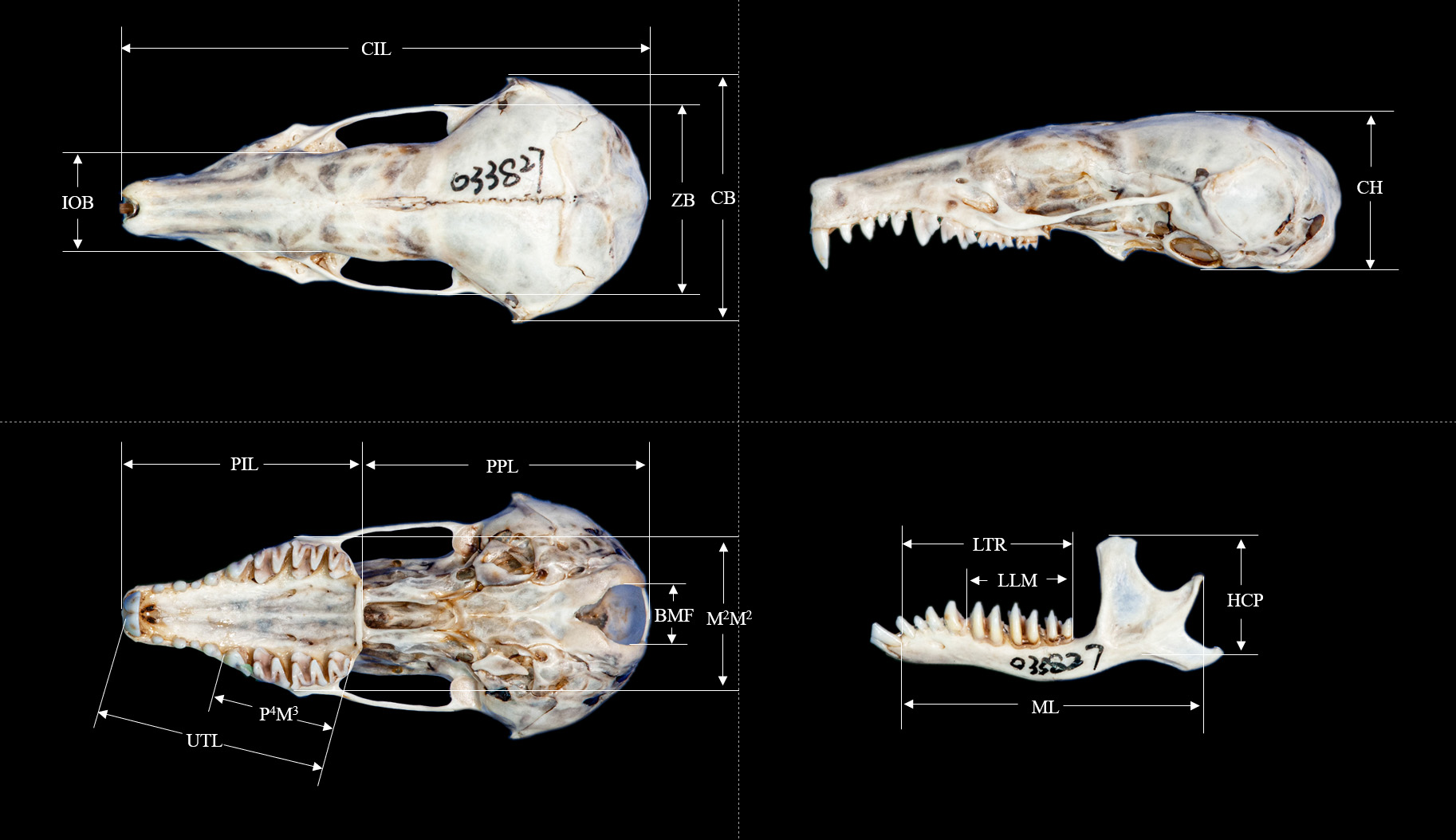

Supplement: gyae142_suppl_Supplementary_Datas_SD2 [file gyae142_suppl_supplementary_datas_sd2.zip › gyae142_suppl_Supplementary_Datas_SD2.jpg]

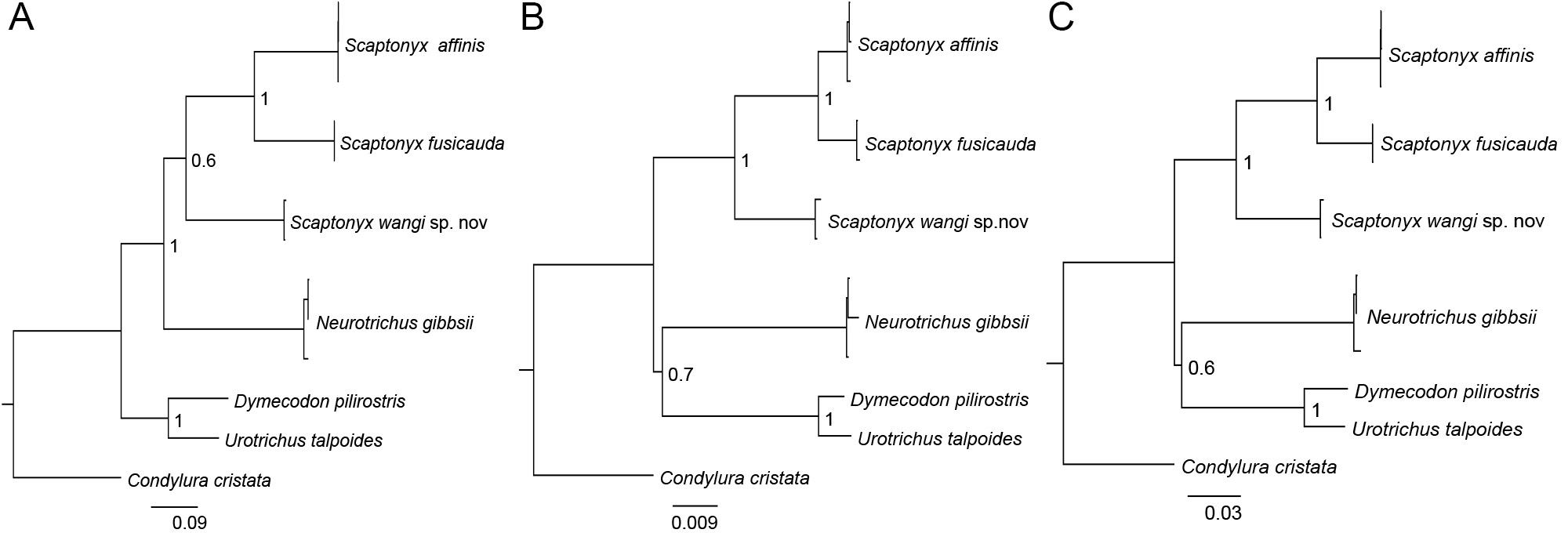

Supplement: gyae142_suppl_Supplementary_Datas_SD8 [file gyae142_suppl_supplementary_datas_sd8.zip › gyae142_suppl_Supplementary_Datas_SD8.jpg]

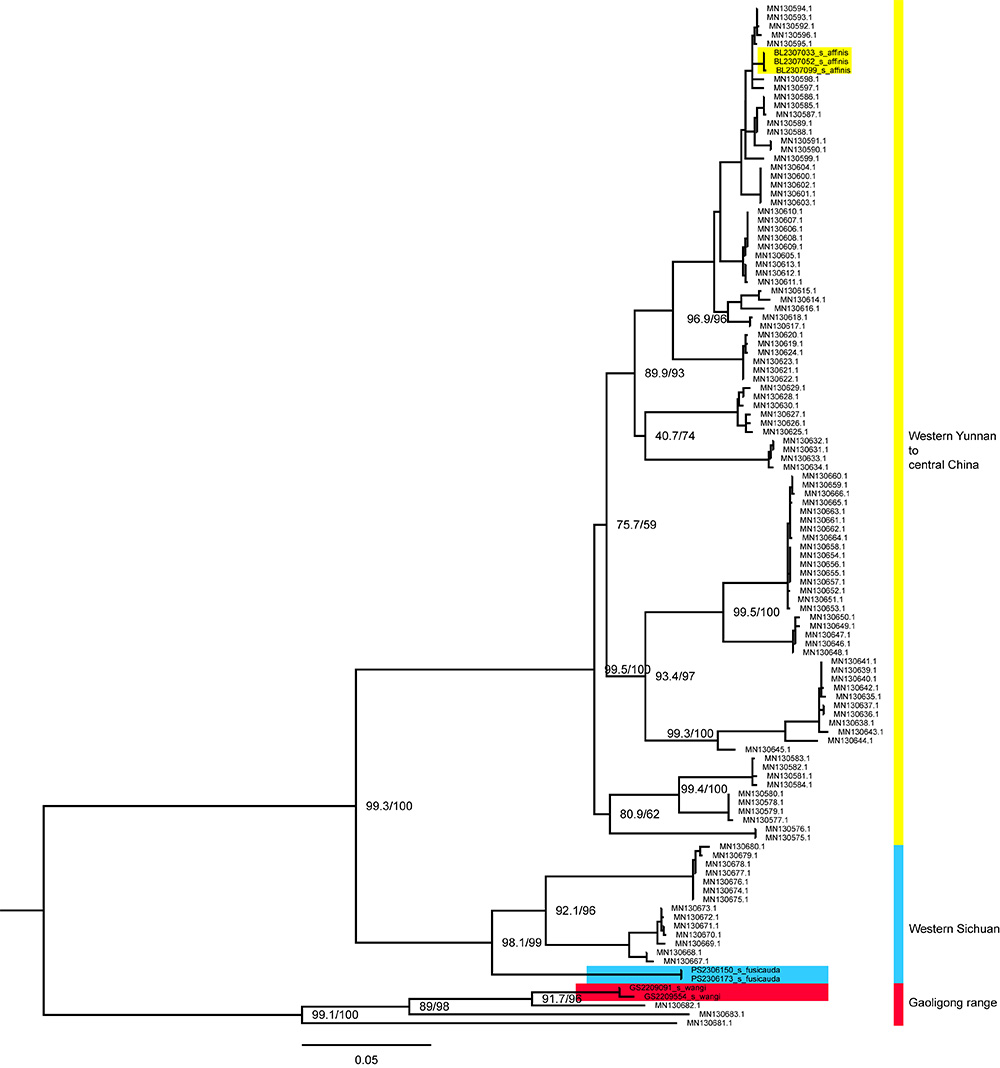

Supplement: gyae142_suppl_Supplementary_Datas_SD9 [file gyae142_suppl_supplementary_datas_sd9.zip › gyae142_suppl_Supplementary_Datas_SD9.jpg]
